# Distribution of Triterpenoids and Steroids in Developing Rugosa Rose (*Rosa* *rugosa* Thunb.) Accessory Fruit

**DOI:** 10.3390/molecules26175158

**Published:** 2021-08-25

**Authors:** Soyol Dashbaldan, Agata Rogowska, Cezary Pączkowski, Anna Szakiel

**Affiliations:** 1 Department of Plant Biochemistry, Institute of Biochemistry, Faculty of Biology, University of Warsaw, 1 Miecznikowa Street, 02-096 Warsaw, Poland; dsoyloo89@gmail.com (S.D.); a.rogowska@biol.uw.edu.pl (A.R.); myhacp@biol.uw.edu.pl (C.P.); 2 School of Industrial Technology, Mongolian University of Science and Technology, 8nd Khoroo, Baga Toiruu 34, Sukhbaatar District, Ulaanbaatar 14191, Mongolia

**Keywords:** achenes, fruit development, fruit ripening, hypanthium, *Rosa rugosa*, rose hip, seeds, steroids, triterpenoids

## Abstract

Triterpenoids and steroids are considered to be important for the fruit quality and health-promoting properties for the consumers. The aim of the study was the determination of the changes in triterpenoid and steroid biosynthesis and the accumulation in hypanthium and achenes of rugosa rose (*Rosa rugosa* Thunb.) hip during fruit development and ripening at three different phenological stages (young fruits, fully developed unripe fruits, and matured fruits). Triterpenoids and steroids were also determined in the peel and the pulp of the matured hips. The obtained results indicated that the distribution of the analyzed compounds in different fruit tissues is a selective process. The increased rate of hydroxylation of triterpenoids, the deposition of hydroxylated acids in fruit surface layer, and the continuous biosynthesis of phytosterols in achenes versus its gradual repression in hypanthium accompanied by the accumulation of their biosynthetic intermediates and ketone derivatives seem to be characteristic metabolic features of maturation of rugosa rose accessory fruit. These observations, apart from providing the important data on metabolic modifications occurring in developing fruits, might have a practical application in defining fruit parts, particularly rich in bioactive constituents, to enable the development of novel functional products.

## 1. Introduction

Triterpenoids and steroids, polycyclic compounds derived from the linear hydrocarbon squalene, are ubiquitously distributed throughout the plant kingdom, including Angiosperms and their fruits [1,2,3]. These compounds are supposed to be important for the fruit quality, particularly due to their biological activities against pathogens, and the influence on the mechanical resistance of the fruit surface [4,5]. Moreover, triterpenoids and steroids present in fruits are of particular interest for the consumers due to their perceived health-promoting attributes. The proper dietary intake of phytosterols is associated with a significant decrease in low-density lipoprotein cholesterol levels [6], whereas the conjugated and non-conjugated triterpenoids are claimed to exert anti-inflammatory, anti-viral, anti-microbial, anti-fungal, and anti-protozoal properties, as well as a number of health beneficial effects, e.g., the activity against cardiovascular disease and diabetes, the ability to suppress bone density loss leading to osteoporosis, and even to inhibit initiation, promotion, and metastasis of cancer [1,7].

Fruit is a specialized complex organ devoted to seed dispersal, and is composed of several interconnected tissues with dedicated roles. The anatomical complexity of fruit is strictly related to the metabolism, as different tissue layers, according to their function, are characterized by a specific distribution and concentration of various metabolites. However, metabolic composition of these tissues is not a stable feature; it undergoes dynamic changes during fruit development and maturation. Fruit can increase in mass or volume by 100-fold or more from fertilization to maturity, thus, as fruit grows, the composition and proportions of the metabolites change dramatically [8,9,10].

**Figure 1:**
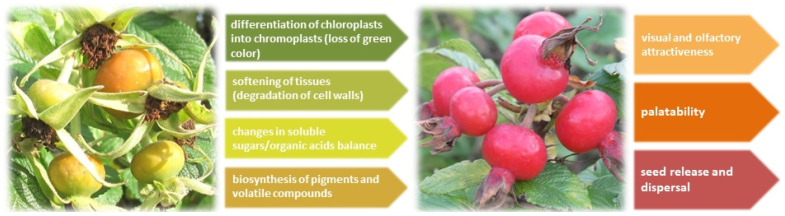
The main processes involved in ripening of edible fruits illustrated by the example of rugosa rose (*Rosa rugosa* Thunb.) accessory fruits referred to as hips.

During ripening of fleshy edible fruits, many metabolic pathways are activated to make the fruit palatable and attractive for potential consumers, simultaneously promoting tissue softening to facilitate seed release [11,12,13]. Some effects of the metabolic changes associated with fruit ripening are easily noticeable, e.g., the transit from partially photosynthetic to heterotrophic status through the parallel differentiation of chloroplasts into chromoplasts, gradual softening of tissues by cell wall-degrading enzymes; the taste attained as a proper balance between sugars and acidic compounds; as well as the production of volatile compounds providing the characteristic flavor (Figure 1). The ripening-associated metabolic pathways are currently the subject of large-scale studies at the transcriptome, proteome, and metabolome levels, since they are fundamental determinants of nutritional and organoleptic value of fruit, also influencing the fruit quality and the duration of post-harvest shelf-life [12,14,15].

One of the plant families bearing fruits with the considerable level of triterpenoids and steroids is *Rosaceae* [16,17]. The pseudo-fruits of *Rosa* species, which are called rose hips, consists of several achenes (real fruits in botanical meaning) enclosed by an enlarged, red, fleshy floral cup (hypanthium). Since the achenes represent separate ripened ovaries derived from a single flower, the entire rose hip can be considered an aggregate fruit or etaerio [18]. The pseudo-fruits from *Rosa* species have been used for alimentation and medicinal purposes (as an herbal remedy for the treatment of various ailments such as flu, colds, inflammations) since ancient times, thanks to their high level content of various nutritional and bioactive compounds exerting health-promoting properties. Currently, medicinal interest in rose hips has significantly increased as a consequence of recent research on their potential application as a treatment for skin disorders, hepatotoxicity, renal disturbances, diarrhea, inflammatory disorders, arthritis, diabetes, hyperlipidaemia, obesity, and cancer [17,19,20]. Rose hips are particularly known as a rich source of ascorbic acid (they are claimed to have the highest content of this vitamin of all popular fruits). However, the significant antioxidant activity of extracts from rose hips is due not only to the high content of vitamin C, but also vitamins B, E, polyphenols, polyunsaturated fatty acids, and carotenoids, which may exert synergistic effects. Another important group of phytochemicals occurring in rose hips are triterpenoids and steroids, although their content in these pseudo-fruits is not as well characterized as the other compounds [19,20,21,22].

*Rosa rugosa* (rugosa rose, beach rose, Japanese rose, or hedgehog rose) is indigenous to eastern Asia. Considered by gardeners as the most beautiful species among wild roses, it was introduced into Europe and North America in the middle of nineteenth century, and generally utilized for ornamental, food, and medicinal purposes [17,20,22]. In some habitats, it is currently considered an invasive species, particularly in seashores of Northern Europe [23]. Being an abundant and widely available plant raw material, it deserves special attention as a source of biologically active phytochemicals with potential medicinal application.

## 2. Results

### 2.1. Identification of Triterpenoids and Steroids

Diethyl ether extracts obtained from dried hypanthium and achenes of rugosa rose pseudo-fruits (accessory fruits) at three consecutive stages of development were fractionated by preparative thin layer chromatography (TLC), as described in Section 4.3. Additionally, the peel of the matured hips and the remaining hypanthium tissues (separated as described in Section 4.1) were also dried and extracted by diethyl ether. All obtained fractions were individually subjected to GC-MS/FID analysis (Section 4.5).

The profile of triterpenoids identified in the present study was similar to the triterpenoid composition determined previously in the rugosa rose hip cuticular wax [16]; however, two additional compounds were detected. Moreover, some significant differences between analyzed fruit parts could be observed. Generally, the triterpenoid profile consisted of pentacyclic compounds of three carbon skeletons: oleanane-, ursane-, and lupane-type, as it can be considered as typical for the majority of Rosaceae plants. The composition of the fraction of neutral triterpenoids, including alcohols, ketones, and aldehydes, consisted of three monols and two diols (the latter not detected previously in rugosa rose cuticular wax [16]), two aldehydes, and one ketone. The ursane-type group of compounds was the most numerous and included α-amyrin (urs-12-en-3β-ol), α-amyrenone (urs-12-en-3-one), uvaol (urs-12-ene-3,28-diol), and ursolic aldehyde (3β-hydroxy-urs-12-en-28-al). The oleanane-type group consisted of β-amyrin (olean-12-en-3β-ol), erythrodiol (olean-12-ene-3,28-diol) and oleanolic aldehyde (3β-hydroxy-olean-12-en-28-al), whereas the lupane-type group was represented by only one compound, lupeol (lup-20 (29)-en-3β-ol). Lupeol formed a common peak with α-amyrin, which had been observed in our previous studies, and their identification in the mixture was confirmed by GC-MS analysis of their authentic standards, examined separately or combined [24].

**Figure 2:**
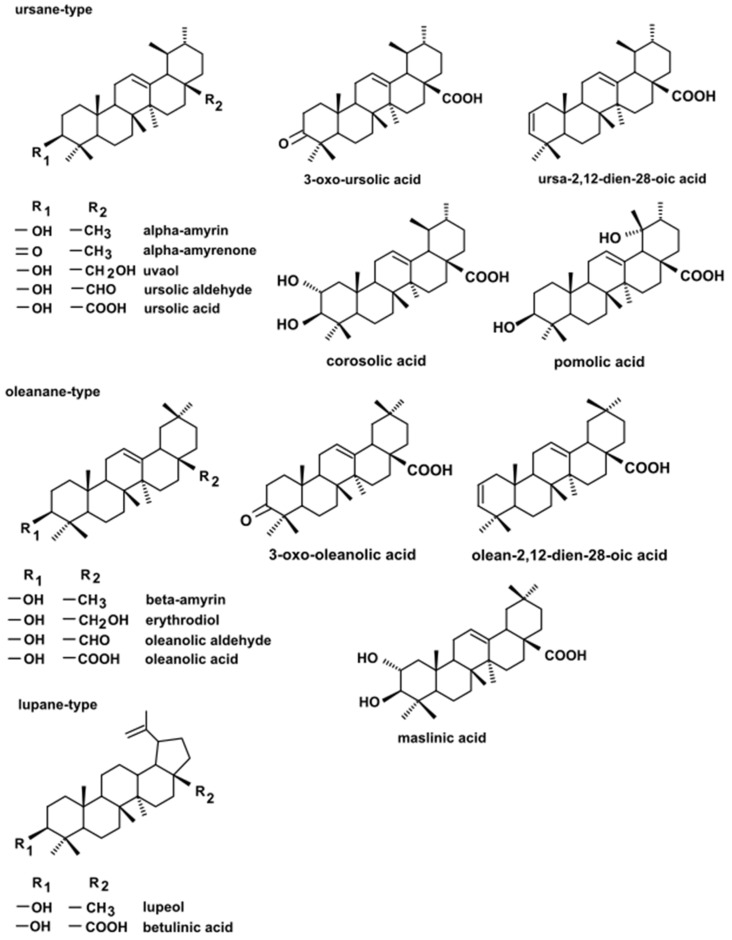
The chemical structures of ursane-, oleanane-, and lupane-type triterpenoids identified in this study.

In turn, the profile of triterpenoid acids was simpler in hypanthium and achenes than in the cuticular waxes. Again, the group of ursane-type compounds was the most numerous, comprising ursolic acid (3β-hydroxy-urs-12-en-28-oic acid), corosolic acid (2α,3β-dihydroxy-urs-12-en-28-oic acid), and pomolic acid (3,19-dihydroxy-urs-12-en-28-oic acid). The oleanane-type group consisted of oleanolic acid (3β-hydroxy-olean-12-en-28-oic acid) and maslinic acid (2α,3β-dihydroxy-olean-12-en-28-oic acid). The lupane-type group was represented again by only one compound, betulinic acid (3β-hydroxy-lup-20 (29)-en-28-oic acid). Additional derivatives of oleanolic and ursolic acids, including 3-oxo-analogs (3-oxo-olean-12-en-28-oic acid and 3-oxo-urs-12-en-28-oic acid) as well as analogs with additional double bond in position 2 (olean-2,12-dien-28-oic acid and ursa-2,12-dien-28-oic acid), identified previously in cuticular waxes [16], were detected exclusively in the extract from the isolated peel, thus confirming their occurrence mainly in the surface layer of rugosa rose hip. The chemical structure of the compounds belonging to the three types of the triterpenoid carbon skeletons are presented in Figure 2.

**Figure 3:**
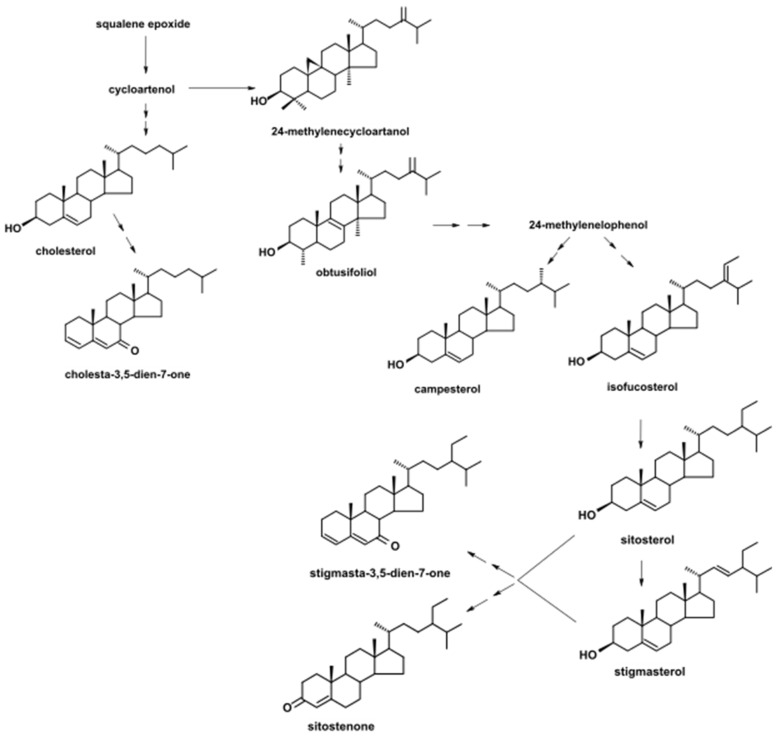
The simplified putative biosynthetic pathway of sterols and steroids in rugosa rose (*Rosa rugosa* Thunb.) accessory fruits. The chemical structures of detected compounds are presented, whereas other important intermediates required in the pathway as branching points are incorporated in the scheme only as names. Both steroid ketones, stigmasta-3.5-dien-7-one and sitostenone, can be derived from sitosterol and stigmasterol.

As in the case of triterpenoids, the main profile of steroids identified in the present study was similar to that detected in the cuticular wax of rugosa rose hip; however, other compounds were also identified. Again, cholesterol (cholest-5-en-3β-ol) as well as several typical sterols characteristic for higher plants were found, featuring campesterol (24*R*-ergost-5-en-3β-ol), isofucosterol (24Z-stigmasta-5,24 (28)-dien-3β-ol, synonym: Δ5-avenasterol), sitosterol (stigmast-5-en-3β-ol), and stigmasterol (22*E*-stigmasta-5,22-dien-3β-ol). However, the profile was enriched in two compounds considered as intermediates of biosynthetic pathway of plant sterols, 24-methylenecycloartanol (24-methylene-9,19-cyclolanostan-3β-ol), and obtusifoliol (24-methylene-29-nor-5α-lanost-8-en-3β-ol, synonym: 4α,14α-dimethyl-5α-ergosta-8,24(28)-dien-3β-ol). Among steroidal derivatives, in addition to stigmasta-3,5-dien-7-one (tremulone) found previously in cuticular waxes, two other ketones were identified: cholesta-3,5-dien-7-one and sitostenone (stigmast-4-en-3-one). Regarding the composition of identified steroids, the presumable pathway of their biosynthesis in developing rugosa rose hips is presented in Figure 3.

### 2.2. Changes in the Content of Triterpenoids and Steroids in Hypanthium during Rugosa Rose Hip Development

The quantification of individual compounds was prepared using an external standard method based on the flame ionization detector (FID) signal as described in Section 4.5. The results obtained for hypanthium (analyzed together with the peel) are presented in Table 1. Due to the coelution of α-amyrin and lupeol, their amounts were quantified together as described in previous studies [3,16,24,25]. 

The total content of triterpenoids and steroids increased more than twice during fruit development from the stage of young hips to the stage of full maturity. Triterpenoid acids were the most prevalent fraction in rugosa rose hypanthium at every stage of hip development, constituting approximately 70% of the total content of triterpenoids and steroids. 

Ursolic acid was the most prominent constituent, ranging from 55% in hypanthium of young hips to 51% in matured hips. The second compound was oleanolic acid, constituting steadily 25% of the fraction of triterpenoid acids during all the period of hip development and maturation. Betulinic acid (lupan-type compound) was much less abundant than the acids of ursane- and oleanane-type, with the amount more than 20-fold lower than ursolic acid and 10-fold lower than oleanolic acid. The most significant process observed for the fraction of triterpenoid acid accumulated in rugosa rose hypanthium was the increase in the content of dihydroxylated acids, particularly pomolic acid, which appeared in the stage of fully developed unripe hips and its level increased almost five-fold during maturation.

The content of neutral triterpenoids in rugosa rose hypanthium increased more than three-fold during hip development and maturation; however, it was still almost six-fold less abundant than the fraction of triterpenoid acids. The prevailing compound was ursolic aldehyde, ranging from 50% of the fraction of neutral triterpenoids in young hips to 36% in matured hips. The most remarkable phenomenon noticed for the fraction of neutral triterpenoids was the absence of diols, erythrodiol, and uvaol in the hypanthium of young hips, their subsequent appearance in fully developed hips, and more than two-fold increase in their content during maturation.

The fraction of steroids constituted 23% of the total content of triterpenoids and steroids in hypanthium of young hips, and decreased to 19% in matured hips, although their amount actually increased 1.7-fold during hip development and maturation. However, this increase in steroid accumulation was not uniform; their content enhanced by 32% during fruit development from the stage of the young hips to the stage of fully developed not ripen hips, and afterwards only by 15% during fruit maturation. Sitosterol was the main phytosterol, ranging from 70% of steroid fraction in young hips to 60% in matured hips. Its accumulation reflected the trend observed for the total steroid fraction, since the amount of sitosterol increased by 30% during fruit development and only by 8% during fruit maturation. The second abundant phytosterol was isofucosterol (approx. 14% of total steroid fraction), followed by campesterol (7%). Apart from the gradual repression of phytosterol accumulation, the increase in the level of their biosynthetic intermediates, i.e., 24-methylenecycloartanol and obtusifoliol, was noticed during hip maturation (by 3.7-fold and almost 2-fold, respectively). Another significant phenomenon was the sharp enhancement of the accumulation of steroid ketones, stigmasta-3,5-dien-7-one (14-fold), sitostenone and cholesta-3,5-dien-7-one (both by 10-fold). Changes in the content of the fractions of triterpenoids and steroids in hypanthium during rugosa rose hip development are shown in Figure S1 (Supplementary Materials).

### 2.3. Changes in the Content of Triterpenoids and Steroids in Achenes during Rugosa Rose Hip Development

The results of the quantification of triterpenoids and steroids accumulated in rugosa rose achenes are presented in Table 2. The total content of the identified compounds increased four-fold during hip development and maturation. The prevailing fraction was steroids, ranging from 85% of the total content of triterpenoids and steroids in achenes of the young hips, to 71% in achenes of matured hips. Triterpenoid acids and neutral triterpenoids were minor fractions during all hip development and maturation.

Among steroids, again sitosterol was the most prominent compound, constituting 80% of all steroids in achenes of young hips and afterwards decreasing to 70% in matured hips. As in hypanthium, the second phytosterol was isofucosterol (10% of the steroid fraction), followed by campesterol (6%). The same phenomena as noticed previously in the case of steroid content in rugosa rose hypanthium were observed, i.e., the gradual accumulation of biosynthetic intermediates and formation of steroid ketone derivatives during the stage of hip maturation. The levels of 24-methylenecycloartanol and obtusifoliol increased by 17% and 37%, respectively, during hip development from the stage of young hips to fully developed hips, and afterwards by 3.5-fold and 4-fold during hip maturation. The amounts of steroid ketones increased sharply during hip maturation, cholesta-3,5-dien-7-one by 6-fold, sitostenone by 3.5-fold, and stigmasta-3,5-dien-7-one by 11.5-fold.

The fraction of triterpenoid acids constituted 8% of the total fraction of triterpenoids and steroids in achenes of young rugosa rose hips and increased to 19% in achenes of matured hips, mainly due to the particularly sharp enhancement of the amount of corosolic acid (31-fold during hip development and maturation). Thus, the profile of the fraction of triterpenoid acids in achenes of the matured hips was different than that in hypanthium, with corosolic acid as the most prominent compound (40% of the fraction), instead of ursolic acid (25%). Another important difference was the ratio of the main acids of ursane-, oleanane-, and lupane-types, reflecting the significantly higher participation of lupane-type betulinic acid. In hypanthium, the ratio of ursolic acid: oleanolic acid: betulinic acid was 18:9:1, whereas in achenes the content of betulinic acid was comparable to that of oleanolic acid, resulting in the ratio of 2.3:1.1:1.

The neutral triterpenoids constituted approximately 8% of the total fraction of triterpenoids and steroids in rugosa rose achenes. The profile of this fraction was also different from that in hypanthium, the main constituent in matured achenes was ursane-type diol, uvaol (30% of the fraction) instead of ursolic aldehyde. Changes in the content of triterpenoids and steroids in achenes (containing seeds) during rugosa rose hip development are shown in Figure S2.

### 2.4. Comparison of the Content of Triterpenoids and Steroids in an Isolated Peel and Remaining Tissues (a Pulp) of Hypanthium of Matured Rosa rugosa Hip

The results of the quantification of triterpenoids and steroids accumulated in the peel of rugosa rose hips and the remaining tissues of hypanthium (the pulp) are presented in Table 3. The profile of triterpenoids and steroids accumulated in the peel is similar to that described previously for rugosa rose hip cuticular waxes [16], although the total content and amounts of individual compounds cannot be directly compared due to their determination in cuticular wax chloroform extract mass. Besides, the peel contains not only cuticular waxes and cuticle, but also the living cells of epidermis.

The distribution of the fractions of steroids, neutral triterpenoids and triterpenoid acids between the peel and the pulp was not equal. As it could be expected, the major fraction accumulated in the peel was the fraction of triterpenoid acids (83% of the total content of triterpenoids and steroids). The content of this fraction was approximately 4-fold higher in the peel than in the pulp, where it constituted 38% of the total content of triterpenoids and steroids. Ursolic acid was the most abundant compound, constituting 39% of the fraction of triterpenoid acids in the peel and 56% in the pulp. It should be noted that dihydroxy acids (corosolic, maslinic and pomolic acids) were accumulated in the peel in significantly higher amounts than in the pulp (constituting together 21% of the fraction of triterpenoid acids in the peel and 16% in the pulp). Moreover, in this experiment, the 3-oxo-analogs and 2,12-dien-analogs of oleanolic and ursolic acids, described previously in cuticular wax [16] but not identified in the extracts of the entire hypanthium in the present study (Table 1), were found.

As in rugosa rose hip cuticular wax, the neutral triterpenoids were the second abundant fraction in the peel. The content of this fraction was approximately equally distributed between the peel and the pulp; however, they constituted only 9% of the total triterpenoids and steroids in the peel, whereas 20% in the pulp. The most prominent compound of this fraction in the peel was ursolic aldehyde (54% of the neutral triterpenoids), whereas uvaol was dominating in the pulp (46%). Indeed, triterpenoid diols, not determined previously in cuticular waxes, seemed to be accumulated almost exclusively in the pulp.

The content of steroids was 2.4-fold higher in the pulp than in the peel. This fraction was the most prevalent in the pulp (42% of the total content of triterpenoids and steroids, whereas it was only 8% in the peel). Sitosterol was the main phytosterol, constituting 71% and 58% of the steroids in the peel and the pulp, respectively. The accumulated intermediates of sterol biosynthesis (24-methylenecycloartanol and obtusifoliol) as well as steroid ketones, apart from stigmasta-3.5-dien-7-one, were found only in the pulp.

## 3. Discussion

Regarding different functions of various fruit tissues, it can be expected that the distribution of specific groups of metabolites is not equal within the fruit. Likewise, the difference in the physical and chemical properties seems to be a logical reason of the disparity in the accumulation of various compounds in fruit tissues, as demonstrated by the localization of the hydrophobic compounds in fruit surface and the soluble substances mainly in fruit flesh [1,9,14]. However, the distribution of metabolites in fruit tissues is not always so evident and easily predictable, particularly when the seeds are also concerned. In pear (*Pyrus communis* L.), the highest concentrations of some phenolic compounds (e.g., polymeric procyanidins) and organic acids (with prevailing malic acid) were found in seeds, lower in peel, and the lowest in fruit pulp [26]. In tomato (*Solanum lycopersicum* L.), starch accumulation was spatially localized in the pericarp and columella, and, surprisingly, the seed locular tissues contained only small amounts of this carbohydrate reserve [9]. The hydrophilic ascorbic acid was accumulated at the highest concentration in the apple (*Malus domestica* Borkh) peel. Moreover, this tissue was capable of *de novo* biosynthesis of ascorbic acid via L-galactose and D-galacturonic acid pathways, whereas the flesh and seeds were only able to synthesize ascorbic acid via L-galactose pathway [27]. The distribution of low polar and hydrophobic triterpenoids and steroids in fruit tissues is also not always predictable; these compounds are abundant in peel but they can be found in other fruit parts, such as flesh and seeds [2,27].

**Figure 4:**
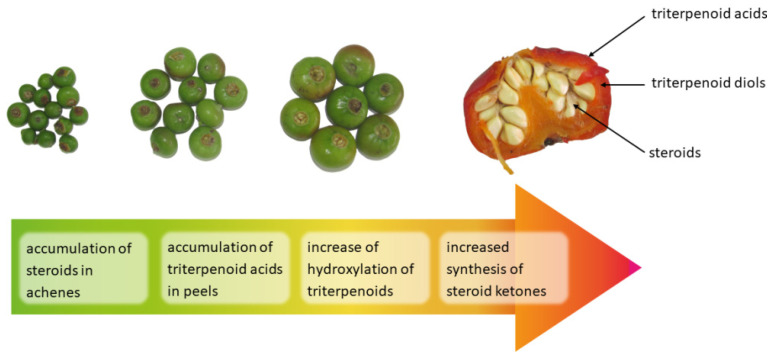
The main differences in accumulation of triterpenoids and steroids in anatomical parts of rugosa rose (*Rosa rugosa* Thunb.) accessory fruits and characteristic processes observed during ripening and maturation.

The present study revealed significant differences in the accumulation of triterpenoids and steroids in analyzed rugosa rose hip tissues and demonstrated some characteristic changes occurring during fruit development, and particularly fruit maturation (Figure 4).

Triterpenoid acids were the most abundant in hypanthium and particularly in the peel, as a consequence of their accumulation in cuticular wax layer [16]. Thus, regarding their partition among different anatomical parts of the fruit, the main target of the transport and accumulation of triterpenoid acids is the peel, including cuticular waxes. However, these compounds also occurred in smaller amounts in the pulp and the achenes, thus indicating that their localization in fruits is not restricted only to the surface, as it was also demonstrated in the case of other fruits, such as pear or Saskatoon berry [2,26]. Nevertheless, in the majority of fruits known for their significant content of triterpenoid acids, the peel remains the best source of these compounds, and various techniques of their efficient extraction are currently the subject of much study [28]. In turn, the relative small amounts of pentacyclic triterpenoids (both acids and alcohols), regarded as defense metabolites, was surprising in rugosa rose achenes, where the accumulation of protective phytochemicals could be expected. Instead, the achenes seem to be particularly rich in steroids and fat oils (mainly fatty acids and long-chain alcohols), constituting the reserve for germination and early period of seedling growth.

The profile of triterpenoid acids was simpler in the pulp and achenes than in the cuticular waxes of rugosa rose hips. The 3-oxo-analogs and 2,12-dien-analogs of oleanolic and ursolic acids were not identified in the extracts of the achenes, the pulp, and even the entire hypanthium. It is consistent with the earlier observation that the analysis of triterpenoid profile in extracts of cuticular waxes or isolated peel is facilitated and more precise in comparison to the analysis of the extracts of entire plant or fruit parts such as hypanthium, due to lower levels of various contaminants [25].

The present study confirmed the previous finding that the maturation of rugosa rose hip is connected with the increased biosynthesis of dihydroxy acids and their accumulation in cuticular waxes [16]. This feature seems to be a part of metabolic program of fruit ripening, leading to substantial changes in fruit surface quality typical for fruit maturation.

The observed significant differences in accumulation of triterpenoids and steroids in anatomical parts of rugosa rose accessory fruit comprise not only an unequal partition of these compounds but various patterns of their composition. For example, diols (erythrodiol and uvaol), not determined previously in cuticular waxes [16], seem to be accumulated almost exclusively in the pulp. Nevertheless, this phenomenon cannot be regarded as universal in fruits, since the significant amounts of triterpenoid diols were found in cuticular waxes of fruits of many other plant species [3,16,24,25]. The profile of triterpenoid acids was significantly different in achenes than in hypanthium, with corosolic acid as the most abundant compound instead of ursolic acid, and the markedly increased level of lupan-type betulinic acid. These observations indicate that the triterpenoid biosynthesis and their consecutive accumulation is strictly controlled in various anatomical parts of the fruit.

The rate of biosynthesis of triterpenoids and steroids was changing significantly during rugosa rose fruit development and maturation. The total content of triterpenoids increased rather constantly in both hypanthium and achenes, with particularly intense biosynthesis of dihydroxy acids and diols during fruit maturation. In turn, while the biosynthesis of steroids was steadily continued in achenes until the end of fruit development, it was significantly hampered during maturation in hypanthium. It markedly influenced the quantitative profile of the biosynthetic intermediates and ketone derivatives in hypanthium, with the first increasing mainly during fruit development and the latter during fruit maturation. The gradual repression of phytosterol biosynthesis in hypanthium seems to be logical, since these compounds are no longer required for membrane formation in new cells when the fruit development is completed; therefore, as a consequence, the excess of intermediates is accumulated mainly in hypanthium (particularly fruit pulp), and the reactions of oxidation begin to modify not only sterols, but probably also various other metabolites during the last stage of fruit maturation.

To our knowledge, this study is the first report on the distribution of triterpenoids and steroids in various anatomical parts of *Rosa rugosa* accessory fruit, including the changes in metabolism and accumulation of these compounds throughout fruit development and maturation. However, since accessory fruits of *Rosa* species are regarded as a valuable source of various health-promoting phytochemicals, the pattern of changes of the content of other compounds (e.g., flavonoids, phenolic acids) in this plant material was previously investigated [29]. Such studies, apart from providing the important data on metabolic modifications occurring in developing fruits, might have a practical application in defining fruit parts particularly rich in bioactive constituents to enable development of novel functional products [2,26]

## 4. Materials and Methods

### 4.1. Plant Material

Accessory fruits of rugosa rose *Rosa rugosa* Thunb. (*Rosaceae*) were collected from a private orchard in Stare Bosewo, central Poland (52°460 N, 21°332 E). Saplings were purchased from the licensed supplier of certified crops, the Polish Vegetable Seed Farming and Nursery enterprise “PNOS”, and cultivated in an open field.

Fruits were collected at three different stages: young fruit (July), fully developed but unripe fruit (August), and mature fruit (September). Replicates of 5–10 intact and healthy fruits were selected from harvested pooled sample sets of approximately 20 fruits per set. At every developmental stage, fruit samples were harvested from the same plant to avoid the impact of plant inter-individual variability. Hips were cut in half with the scalpel, and achenes were carefully separated from hypanthium. The additional samples of matured hips were gently peeled to separate the peel from the remaining tissues of hypanthium, referred to as pulp. The peel of the matured rugosa rose hips is clearly distinguished and it can be easily detached from the rest of the hip (Figure S3). The obtained samples of individual fruit components were dried and powdered in a laboratory mill. All experiments were performed in three replicates.

### 4.2. Chemicals and Standards

All solvents used for extraction and analysis, i.e., chloroform, diethyl ether, and methanol (purchased from Chempur, Piekary Śląskie, Poland), were of analytical grade. Authentic standards of α-amyrin and ursolic acid methyl ester were purchased from Roth (Karlsruhe, Germany), and β-amyrin, lupeol, uvaol, betulinic acid, oleanolic acid, campesterol, sitosterol, and stigmasterol were purchased from Sigma-Aldrich (Steinheim, Germany).

### 4.3. Extraction and Fractionation

#### 4.3.1. Extraction

The obtained dried samples of rugosa rose hip components (entire hypanthium, achenes, separated peels, and the remaining tissues of hypanthium, referred to as pulp) were extracted with diethyl ether in a Soxhlet apparatus during 10 h. Extracts were evaporated with the use of vacuum rotary evaporator. The extracts obtained from hypanthium, peels, and pulp yielded dry residue after evaporation, whereas the concentrated extracts from achenes were oily.

#### 4.3.2. Fractionation of Diethyl Ether Extracts

Evaporated diethyl ether extracts were fractionated by adsorption preparative TLC on 20 cm × 20 cm glass plates coated manually with silica gel 60H (Merck, Darmstad, Germany). The solvent system, chloroform:methanol 97:3 (*v*/*v*), was applied for developing. The fractions of triterpenoid acids and free (non-esterified) steroids and neutral triterpenoids were localized on the plates by comparison to the appropriate standards, as described earlier [16,24]. Fractions were eluted from the gel in diethyl ether. Subsequently, fractions containing free neutral triterpenes and sterols (*R*_F_ 0.3–0.9) were directly analyzed by GC-MS, whereas the fractions containing triterpene acids (*R*_F_ 0.2–0.3) were methylated with diazomethane. The average recovery of α-amyrin, uvaol, stigmasterol, and ursolic acid methyl ester from preparative TLC plates was 98.6, 97.2, 98.9, and 96.1%, respectively [25].

### 4.4. Derivatization of Triterpenoid Acids

Nitrosomethylurea (2.06 g) was added to a mixture of 20 mL of diethyl ether and 6 mL of 50% aqueous KOH, and the organic layer was then separated from the aqueous layer. Samples containing triterpenoid acids were dissolved in 2 mL of the obtained solution of diazomethane in diethyl ether, and held at 2 °C for 24 h.

### 4.5. Identification and Quantification of Steroids and Triterpenoids by GC-MS/FID

An Agilent Technologies 7890A gas chromatograph (Perlan Technologies, Warszawa, Poland) equipped with a 5975C mass spectrum detector was used for qualitative and quantitative analyses. Samples dissolved in diethyl ether:methanol (5:1, *v*/*v*) were applied (in a volume of 1–4 μL) using 1:10 split injection. The column used was a 30 m × 0.25 mm-i.d., 0.25-μm HP-5MS UI (Agilent Technologies, Santa Clara, CA, USA). Helium was used as the carrier gas at a flow rate of 1 mL/min. The separation was made with the following temperature program: initial temperature of 160 °C held for 2 min, then increased to 280 °C at 5 °C/min, and the final temperature of 280 °C held for a further 44 min. The other employed parameters were as follows: inlet and FID (flame ionization detector, part of 7890A chromatograph) temperature of 290 °C; MS transfer line temperature of 275 °C; quadrupole temperature of 150 °C; ion source temperature of 230 °C; EI of 70 eV; *m*/*z* range of 33–500; FID gas (H_2_) flow of 30 mL·min^−1^ (hydrogen generator HydroGen PH300, Peak Scientific, Inchinnan, UK); and air flow of 400 mL·min^−1^. Individual compounds were identified by comparing their mass spectra with spectral libraries (Wiley 9th ED. and NIST 2008 Lib. SW Version 2010), with previously reported data and the results of earlier experiments, as well as by comparison of their retention times and corresponding mass spectra with those of authentic standards, where available. Thus, identification of α- and β-amyrins, lupeol, uvaol, methyl esters of betulinic, oleanolic and ursolic acids, campesterol, sitosterol, and stigmasterol was made by comparing their mass spectra with spectral libraries and mass spectra of authenthic standards, as well as their chromatographic properties, i.e., retention times. The identification of other compounds was made on the base of spectral libraries and data from references cited therein. Quantitation of individual compounds was conducted with a FID detector and performed using an external standard method based on calibration curves determined for authentic standards of ursolic acid methyl ester, α-amyrin, and stigmasterol [3,16].

### 4.6. Statistical Analysis of Data

All experiments were performed in triplicate. Data were presented as the means ± standard deviation of three independent samples analyzed in triplicate. The data were subjected to one-way analysis of variance (ANOVA), and the differences between means were evaluated using Duncan’s multiple-range test. Statistical significance was considered to be obtained at *p* < 0.05.

## 5. Conclusions

The obtained results revealed significant differences in biosynthesis and accumulation of triterpenoids and steroids in various anatomical parts of rugosa rose accessory fruit throughout fruit development and maturation. These findings indicate that the distribution of the analyzed compounds in different fruit tissues is a selective process, i.e., the triterpenoid and steroid content of the fruit peel including cuticular waxes, or achenes containing seeds, is not a simple reflection of the composition of these compounds in hypanthium. The biosynthesis and accumulation of triterpenoids and steroids seem to be specifically regulated in order to fulfill the functions of various fruit parts, and integrated within the program of fruit growth. The rhythm of the observed metabolic changes is coordinated with consecutive stages of fruit development and maturation. Different phenomena, such as the increased rate of hydroxylation of triterpenoids, the deposition of hydroxylated acids in fruit surface layer, and the continuous biosynthesis of phytosterols in achenes versus its gradual repression in hypanthium accompanied by the accumulation of their biosynthetic intermediates and ketone derivatives, seem to be a characteristic metabolic features of maturation of rugosa rose accessory fruit.

## Figures and Tables

**Table 1:** Content of triterpenoids and steroids in hypanthium during rugosa rose (*Rosa rugosa*) fruit development.

Compound	Content [µg/g d.w. ± SD (Standard Deviation)]		
	Young Fruit	Fully Developed Green Fruit	Ripened Fruit
α-amyrin/lupeol	5.64 ± 0.62 ^a^	7.32 ± 0.84 ^b^	10.06 ± 1.08 ^c^
β-amyrin	4.08 ± 0.43 ^a^	6.12 ± 0.66 ^b^	8.51 ± 0.79 ^c^
α-amyrenone	0.06 ± 0.01 ^a^	1.23 ± 0.20 ^b^	2.48 ± 0.32 ^c^
oleanolic aldehyde	6.58 ± 0.72 ^a^	18.75 ± 2.01 ^b^	26.63 ± 2.80 ^c^
ursolic aldehyde	16.13 ± 1.55 ^a^	38.64 ± 4.08 ^b^	52.06 ± 6.54 ^b^
erythrodiol	n.d.	4.05 ± 0.39 ^a^	8.82 ± 0.94 ^b^
uvaol	n.d.	18.64 ± 1.92 ^a^	35.98 ± 4.12 ^b^
Sum of neutral triterpenoids	32.49	94.75	144.54
oleanolic acid	109.14 ± 15.02 ^a^	168.95 ± 18.11 ^b^	212.62 ± 24.84 ^c^
ursolic acid	234.56 ± 25.14 ^a^	361.08 ± 40.30 ^b^	431.95 ± 48.01 ^c^
betulinic acid	10.82 ± 1.24 ^a^	17.44 ± 2.06 ^b^	23.97 ± 2.50 ^c^
maslinic acid	n.d.	n.d.	4.12 ± 0.48
corosolic acid	66.71 ± 7.15 ^a^	98.02 ± 10.40 ^b^	133.85 ± 15.03 ^c^
pomolic acid	n.d.	6.46 ± 0.72 ^a^	30.80 ± 3.35 ^b^
Sum of triterpenoid acids	421.23	651.95	837.31
campesterol	10.05 ± 1.20 ^a^	14.02 ± 1.66 ^b^	16.31 ± 2.04 ^b^
cholesterol	1.64 ± 0.18 ^a^	3.19 ± 0.35 ^b^	3.86 ± 0.41 ^b^
cholesta-3,5-dien-7-one	0.23 ± 0.03 ^a^	0.62 ± 0.18 ^b^	2.18 ± 0.22 ^c^
isofucosterol	19.82 ± 2.12 ^a^	28.24 ± 3.06 ^b^	31.49 ± 4.12 ^b^
24-methylenecycloartanol	2.17 ± 0.25 ^a^	6.75 ± 0.71 ^b^	8.13 ± 0.95 ^b^
obtusifoliol	0.68 ± 0.07 ^a^	1.12 ± 0.20 ^b^	1.24 ± 0.10 ^b^
sitosterol	95.94 ± 10.16 ^a^	132.36 ± 10.84 ^b^	142.42 ± 18.82 ^b^
sitostenone	0.82 ± 0.09 ^a^	2.85 ± 0.31 ^b^	8.18 ± 0.44 ^c^
stigmasterol	3.81 ± 0.43 ^a^	5.02 ± 0.48 ^b^	5.33 ± 0.61 ^b^
stigmasta-3.5-dien-7-one	1.06 ± 0.92 ^a^	4.23 ± 0.50 ^b^	14.92 ± 1.24 ^c^
Sum of steroids	136.22	198.40	234.06
Total	589.94	945.10	1215.91

Results are referenced to hypanthium dry weight and expressed as the mean ± SD of three independent samples analyzed in triplicate. Results in rows not sharing a common letter are significantly different (*p* < 0.05).

**Table 2:** Content of triterpenoids and steroids in achenes during rugosa rose (*Rosa rugosa*) fruit development.

Compound	Content [µg/g d.w. ± SD (Standard Deviation)]		
	Young Fruit	Fully Developed Green Fruit	Ripened Fruit
α-amyrin/lupeol	1.42 ± 0.16 ^a^	3.41 ± 0.36 ^b^	6.96 ± 0.72 ^c^
β-amyrin	0.68 ± 0.08 ^a^	1.13 ± 0.15 ^b^	2.51 ± 0.30 ^c^
oleanolic aldehyde	1.26 ± 0.16 ^a^	2.13 ± 0.30 ^b^	3.91 ± 0.45 ^c^
ursolic aldehyde	3.18 ± 0.42 ^a^	5.06 ± 0.62 ^b^	8.25 ± 1.01 ^c^
erythrodiol	0.42 ± 0.05 ^a^	1.38 ± 0.20 ^b^	3.66 ± 0.42 ^c^
uvaol	1.04 ± 0.08 ^a^	2.80 ± 0.32 ^b^	10.58 ± 1.24 ^c^
Sum of neutral triterpenoids	8.00	15.91	35.87
oleanolic acid	1.75 ± 0.20 ^a^	7.03 ± 0.68 ^b^	10.33 ± 1.15 ^c^
ursolic acid	4.06 ± 0.42 ^a^	15.81 ± 2.05 ^b^	21.68 ± 2.54 ^c^
betulinic acid	1.57 ± 0.13 ^a^	6.66 ± 0.74 ^b^	9.25 ± 1.04 ^c^
maslinic acid	n.d.	0.29 ± 0.03 ^a^	1.71 ± 0.20 ^b^
corosolic acid	1.04 ± 0.01 ^a^	10.52 ± 1.20 ^b^	32.38 ± 3.56 ^c^
pomolic acid	n.d.	2.15 ± 0.19 ^a^	6.04 ± 0.68 ^b^
Sum of triterpenoid acids	8.42	42.46	81.39
campesterol	4.13 ± 0.39 ^a^	7.04 ± 0.82 ^b^	18.83 ± 2.05 ^c^
cholesterol	0.25 ± 0.03 ^a^	0.53 ± 0.06 ^b^	1.14 ± 0.20 ^c^
cholesta-3,5-dien-7-one	0.03 ± 0.01 ^a^	0.08 ± 0.02 ^b^	0.51 ± 0.07 ^c^
isofucosterol	7.08 ± 0.82 ^a^	13.72 ± 1.54 ^b^	31.12 ± 3.58 ^c^
24-methylenecycloartanol	2.24 ± 0.32 ^a^	3.05 ± 0.41 ^b^	10.83 ± 1.01 ^c^
obtusifoliol	0.75 ± 0.08 ^a^	1.20 ± 0.13 ^b^	4.88 ± 0.4:’;2 ^c^
sitosterol	72.92 ± 10.12 ^a^	140.46 ± 18.88 ^a^	208.08 ± 30.02 ^b^
sitostenone	1.21± 0.02 ^a^	2.15 ± 0.27 ^b^	7.66 ± 0.62 ^c^
stigmasterol	1.57 ± 0.15 ^a^	2.93 ± 0.31 ^b^	6.11 ± 0.65 ^c^
stigmasta-3.5-dien-7-one	0.34 ± 0.03 ^a^	0.58 ± 0.06 ^b^	6.66 ± 0.72 ^c^
Sum of steroids	90.42	171.74	295.82
Total	106.84	230.11	413.08

Results are referenced to achenes dry weight and expressed as the mean ± SD of three independent samples analyzed in triplicate. Results in rows not sharing a common letter are significantly different (*p* < 0.05).

**Table 3:** Content of triterpenoids and steroids in isolated peel and remaining tissues of hypanthium of matured *Rosa rugosa* hip.

Compound	Content [µg/g d.w. ± SD (Standard Deviation)]	
	Peel	Remaining Hypanthium Tissues (Pulp)
α-amyrin/lupeol	6.81 ± 0.75 ^a^	3.02 ± 0.38 ^b^
β-amyrin	5.78 ± 0.62 ^a^	2.89 ± 0.31 ^b^
α-amyrenone	1.63 ± 0.18 ^a^	0.83 ± 0.10 ^b^
oleanolic aldehyde	19.45 ± 2.01 ^a^	5.08 ± 0.52 ^b^
ursolic aldehyde	40.67 ± 4.55 ^a^	11.43 ± 1.30 ^b^
erythrodiol	n.d.	8.54 ± 1.02
uvaol	1.27 ± 0.02 ^a^	34.86 ± 4.04 ^b^
Sum of neutral triterpenoids	75.61	76.65
oleanolic acid	169.45 ± 20.30 ^a^	40.09 ± 4.81 ^b^
ursolic acid	262.66 ± 32.08 ^a^	91.86 ± 10.56 ^b^
betulinic acid	20.17 ± 2.55 ^a^	4.03 ± 0.51 ^b^
3-oxo-oleanolic acid	0.02 ± 0.01	n.d.
3-oxo-ursolic acid	0.49 ± 0.02	n.d.
olean-2,12-dien-28-oic acid	0.28 ± 0.01	n.d.
ursa-2,12-dien-28-oic acid	1.61 ± 0.20	n.d.
maslinic acid	3.80 ± 0.42 ^a^	0.37 ± 0.02 ^b^
corosolic acid	110.69 ± 15.01 ^a^	22.15 ± 3.09 ^b^
pomolic acid	28.43 ± 3.15 ^a^	4.66 ± 0.54 ^b^
Sum of triterpenoid acids	679.60	162.16
campesterol	1.09 ± 0.12 ^a^	16.14 ± 2.08 ^b^
cholesterol	1.42 ± 0.16 ^a^	2.38 ± 0.34 ^b^
cholesta-3,5-dien-7-one	n.d.	2.04 ± 0.26
isofucosterol	9.56 ± 1.88 ^a^	20.13 ± 3.01 ^b^
24-methylenecycloartanol	n.d.	8.85 ± 1.01
obtusifoliol	n.d.	1.12 ± 0.18
sitosterol	51.92 ± 6.44 ^a^	100.41 ± 12.05 ^b^
sitostenone	n.d.	7.90 ± 8.55
stigmasterol	1.98 ± 0.22 ^a^	3.42 ± 0.44 ^b^
stigmasta-3.5-dien-7-one	6.85 ± 0.80 ^a^	9.27 ± 1.15 ^b^
Sum of steroids	72.82	171.66
Total	828.03	410.47

Results are referenced to dry weight of peel and pulp, respectively, and expressed as the mean ± SD of three independent samples analyzed in triplicate. Results in rows not sharing a common letter are significantly different (*p* < 0.05).

## Supplementary Materials

The following are available online. Table S1. Relative retention times and characteristic ions of mass spectra of identified steroids and triterpenoids. Figure S1. Changes in the content of triterpenoids and steroids in hypanthium during rugosa rose (*Rosa rugosa)* fruit development. Figure S2. Changes in the content of triterpenoids and steroids in achenes (containing seeds) during rugosa rose (*Rosa rugosa)* fruit development. Figure S3. The peels isolated from the matured *Rosa rugosa* hips and the remaining hips without peel.

## Sample Availability

Samples of the compounds are not available from the authors.
