# Supplementary material for: Distribution of Triterpenoids and Steroids in Developing Rugosa Rose (Rosa rugosa Thunb.) Accessory Fruit
Source: Molecules. 2021 Aug 25;26(17):5158. doi: 10.3390/molecules26175158 (PMC8433923; doi:10.3390/molecules26175158)
Supplement: Supplementary file 1 [file molecules-26-05158-s001.zip › molecules-1352066-supplementary.pdf]

Distribution of triterpenoids and steroids in different anatomical parts of rugosa rose hip (*Rosa rugosa* Thunb.) during development and ripening

Table S1. Retention times and characteristic ions of mass spectra of identified steroids and triterpenoids

**Retention time (min)**	**Compound**	**Mass spectrum *m/z* (relative intensity)**
31.6	Cholesterol	386 (26), 107 (50), 105 (48), 91 (57), 81 (54), 79 (46), 69 (47), 57 (87), 55 (73), 43 (100), 41 (55)
33.5	Cholesta-3,5-dien-7-one	382 (54), 187 (25), 175 (18), 174 (100), 161 (29), 159 (20), 81 (17), 55 (16), 43 (20), 41 (18)
34.2	Campesterol	400 (30), 107 (51), 105 (55), 95 (49), 83 (45), 81 (64), 71 (62), 57 (77), 55 (77), 43 (100), 41 (52)
35.1	Stigmasterol	412 (36), 145 (64), 107 (52), 95 (100), 83 (66), 81 (90), 78 (60), 69 (67), 67 (85), 55 (69)
36.0	Obtusifoliol	426 (41), 412 (34), 411 (100), 245 (24), 109 (24), 95 (30), 81 (21), 69 (34), 55 (36), 41 (20)
36.9	Sitosterol	414 (29), 145 (54), 107 (59), 105 (60), 95 (54), 91 (49), 81 (57), 57 (68), 55 (70), 43 (100), 41 (44)
38.1	β-Amyrin	426 (27), 219 (18), 218 (100), 203 (49), 189 (17), 135 (11), 109 (13), 105 (12), 95 (15), 81 (18), 69 (14)
38.6	α-Amyrenone	424 (12), 219 (19), 218 (100), 203 (24), 189 (16), 135 (19), 133 (18), 122 (18), 119 (17), 95 (16), 55 (18)
39.2	α-Amyrin/	426 (4), 218 (100), 203 (20), 189 (36), 135 (35), 121 (32), 109 (32), 107 34), 95 (40), 81 (33), 55 (31)
	Lupeol	426 (18), 207 (67), 189 (90), 135 (83), 121 (80) 109 (85), 121 (80), 95 (100), 93 (87), 81 (86),
39.7	Stigmasta-3,5-dien-7-one	410 (32), 187 (27), 174 (100), 161 (37), 159 (26), 91 (28), 57 (28), 55 (37), 43 (44), 41 (28)
41.2	24-methylenecycloartanol	440 (5), 121 (60), 119 (55), 109 (62), 107 (76), 105 (57), 95 (98), 93 (64), 81 (72), 69 (99), 55 (100)
42.0	Sitostenone	440 (5), 121 (60), 119 (55), 109 (62), 107 (76), 105 (57), 95 (98), 93 (64), 81 (72), 69 (99), 55 (100)
47.8	Oleanolic aldehyde	440 (2), 232 (28), 207 (20), 204 (39), 203 (100), 189 (29), 105 (18), 81 (19), 69 (20), 55 (29)
51.6	Ursolic aldehyde	440 (1), 207 (26), 204 (23), 203 (100), 133 (42), 119 (18), 105 (18), 95 (18), 81 (18), 55 (18), 43 (20)
52.1	Erythrodiol	442 (1), 204 (17), 203 (100), 133 (7), 119 (9), 105 (8), 95 (9), 93 (8), 81 (8), 69 (9), 55 (8)
55.8	Uvaol	442 (1), 207 (13), 204 (17), 203 (100), 133 (33), 119 (13), 105 (11), 95 (12), 81 (10), 69 (10), 55 (11)
Free acids analyzed after methylation:		
41.9	Olean-2,12-dien-28-oic acid methyl ester	452(11), 425 (9), 263 (11), 262 (61), 221 (14), 203 (100), 190 (15), 189 (22), 133 (14), 119 (12)

44.7	3-Oxo-olean-12-en-28-oic acid methyl ester	468 (6), 262 (32), 204 (17), 203 (100), 202 (21), 189 (29),133 (17), 119 (14), 105 (12), 55 (12)
46.6	Oleanolic acid methyl ester	470 (1), 262 (48), 207 (13), 204 (16), 203 (100), 202 (21), 189 (22), 133 (17), 119 (13), 105 (14)
46.9	Betulinic acid methyl ester	470 (5), 207 (41), 203 (38), 189 (100), 175 (40), 119 (41), 107 (38), 105 (37), 95 (37), 93 (38)
47.2	3-Oxo-urs-12-en-28-oic acid methyl ester	468 (3), 263 (21), 262 (96), 249 (20), 204 (17), 203 (100), 189 (29), 133 (79), 119 (30), 105 (19)
49.7	Ursolic acid methyl ester	470 (1), 263 (20), 262 (100), 207 (32), 203 (93), 189 (29), 133 (76), 119 (34), 105 (21), 95 (18)

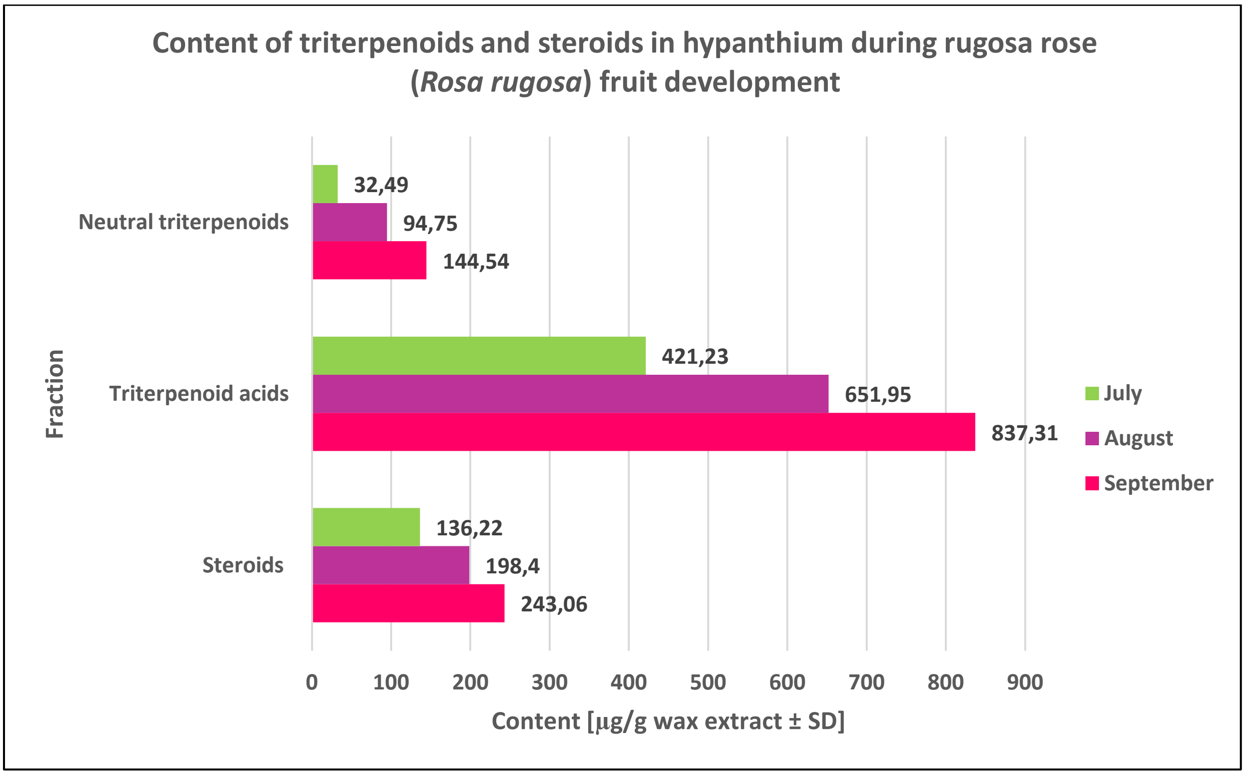

Figure S1. Changes in the content of triterpenoids and steroids in hypanthium during rugosa rose (*Rosa rugosa)* fruit development. July – the stage of young fruits, August – the stage of fully developed unripe fruits, Septemberv – the stage of matured fruits.

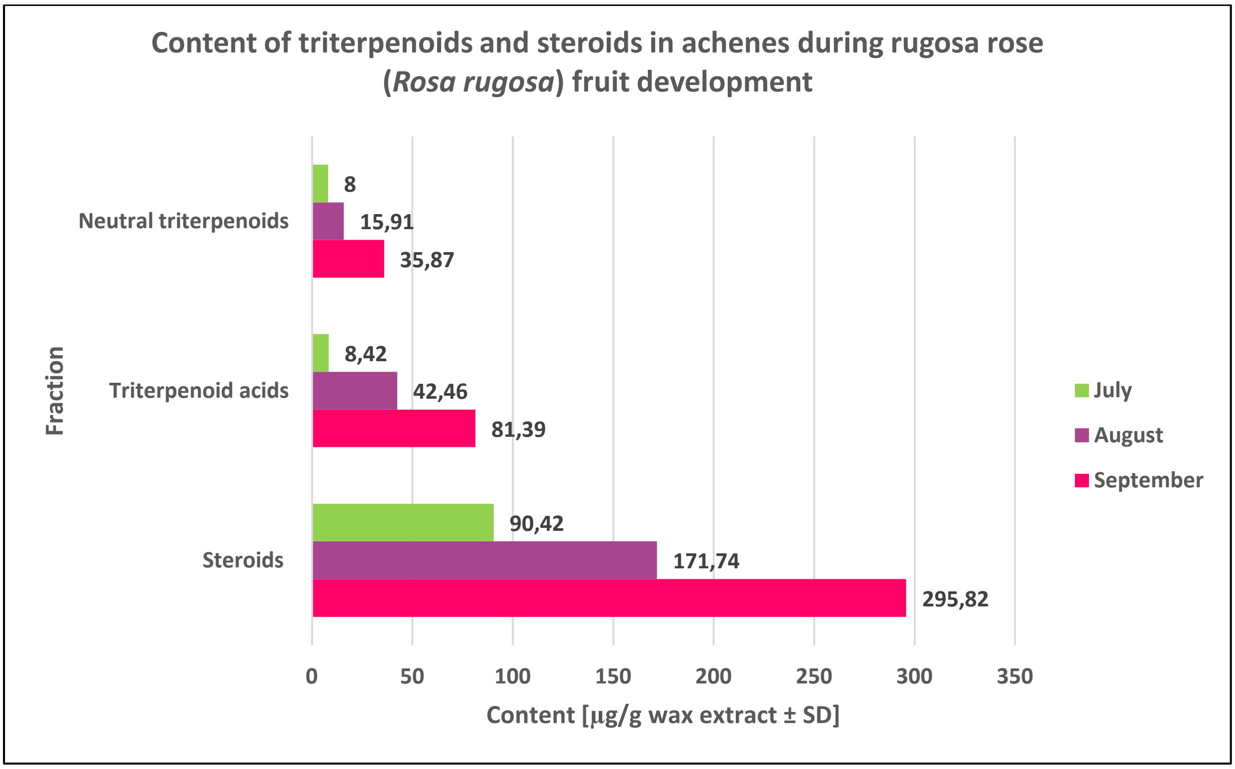

Figure S2. Changes in the content of triterpenoids and steroids in achenes (including seeds) during rugosa rose (*Rosa rugosa)* fruit development. July – the stage of young fruits, August – the stage of fully developed unripe fruits, Septemberv – the stage of matured fruits.

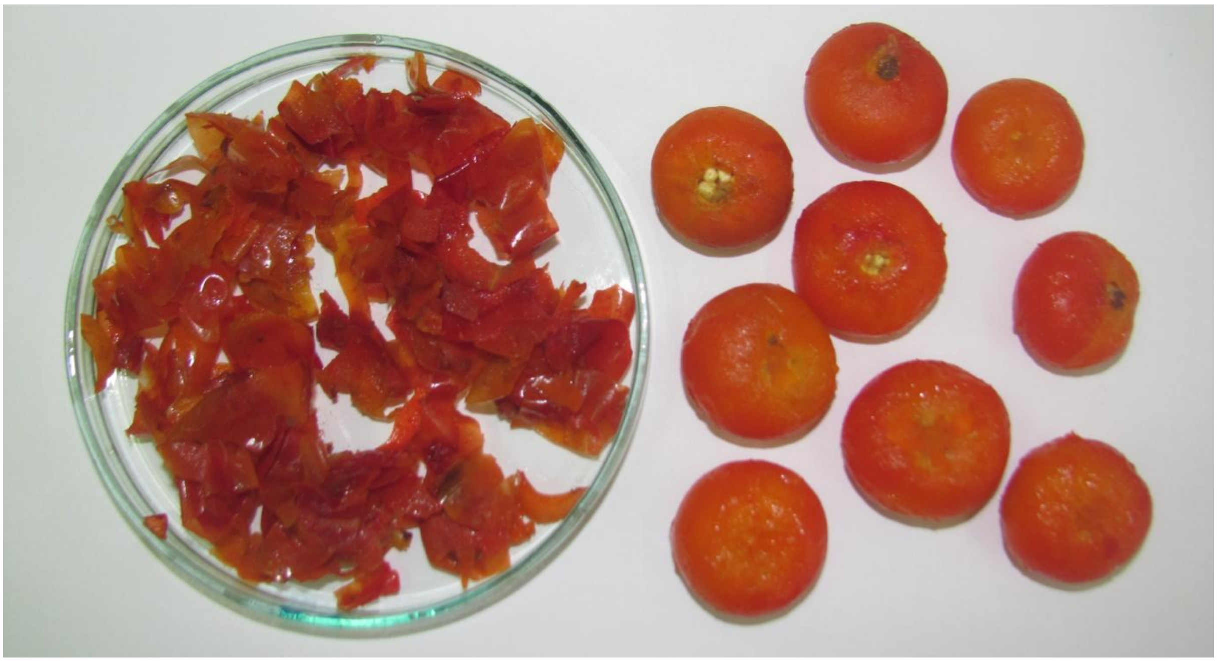

Figure S3. The peels isolated from the matured rosa rugosa hips (on Petri dish) and the remaining hips without peel (on the right).
